# Antimicrobial peptides: emerging next-generation strategy for sustainable plant disease management

**DOI:** 10.3389/frabi.2026.1766594

**Published:** 2026-03-25

**Authors:** Dipayan Das, Tasqeen Khan, Jinkee Kalita, Sarvesh Rustagi, Sujogya Kumar Panda, Niraj Singh, Yugal Kishore Mohanta

**Affiliations:** 1 Department of Microbiology, Royal School of Biosciences, The Assam Royal Global University, Guwahati, Assam, India; 2 Department of Food Technology, School of Agriculture, Dev Bhoomi Uttarakhand University, Dehradun, Uttarakhand, India; 3 Centre for Biotechnology, Shiksha “O” Anusandhan (Deemed to be University), Bhubaneswar, Odisha, India; 4 Nano-biotechnology and Translational Knowledge Laboratory, Department of Applied Biology, University of Science and Technology Meghalaya, Techno City, Baridua, Meghalaya, India

**Keywords:** antibiotics, biocontrol agents, plant immunity, plant pathogens, sustainable agriculture, synthetic biology

## Abstract

Plant diseases reduce agricultural productivity worldwide, and this decline is further accelerated by climate variability, monoculture cultivation systems, and the excessive use of synthetic agrochemicals. Overuse of chemical (synthetic) pesticides in agriculture results in ecological stress, including loss of beneficial microbes. As a solution, antimicrobial peptides (AMPs) are viable natural alternative to antibiotics and pesticides, due to their potent, broad-spectrum, and targeted properties, as well as their low susceptibility to the development of resistance. As small cationic amphipathic molecules found in plants, animals, and microorganisms, these AMPs are known to modulate membrane permeabilisation, disrupt intracellular systems, and stimulate the immune response. The AMP defence system depends on the highly interconnected gene network that supports efficient signal transmission and tightly coordinated gene clusters that support systematic responses to pathogen attack. These molecules can be considered as attractive biocidal agents due to their ability to target microbial membranes and cause rapid cell death, thereby having potential as broad-spectrum biocontrol agents against bacteria, fungi, and viruses. AMPs are also effective against multidrug-resistant pathogens. In plants, AMP families such as defensins, thionins, cyclotides, LTPs (lipid transfer proteins), snakins, and hevein-like peptides act as constitutive “natural antibiotics” which are involved in activating defensive signalling cascades upon pathogen infection. Microbial AMPs, such as bacteriocins, suppress pathogenic and spoilage bacteria by forming pores and inhibiting cell wall synthesis. At the same time, lipopeptides promote beneficial biofilms and plant defence pathways without direct toxicity. Progress in molecular biology, computational modelling, and synthetic biology has revealed the discovery, engineering, and optimisation of AMPs for agriculture. This review summarises the mechanisms of antibiotic mimicry by AMPs and discusses their structural and functional diversity, as well as their potential applications in sustainable plant disease management. The present study also evaluated AMPs as an alternative to chemical pesticides and antimicrobial agents, offering an environmentally compatible, durable, and efficient approach to preventing plant diseases.

## Graphical Abstract

**Figure:**
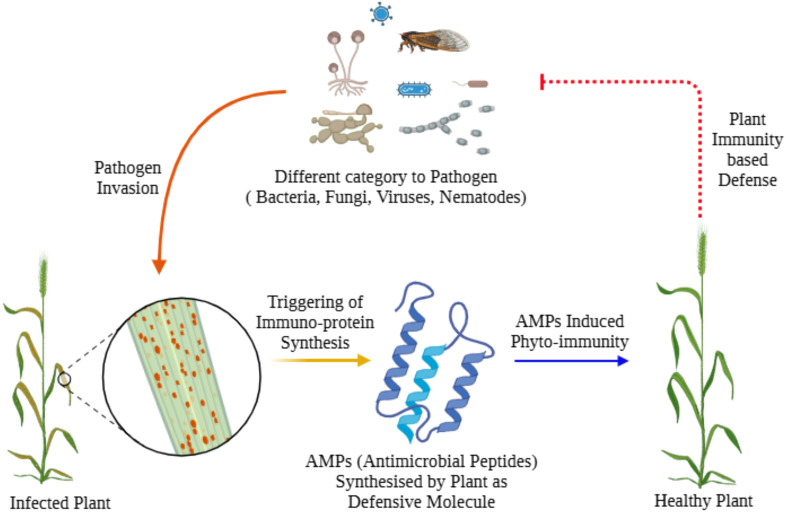

## 1 Introduction

Plant diseases exhibit a significant threat to global agricultural productivity, with their prevalence and severity intensified by climate variability, monoculture-based cropping systems, and the excessive use of synthetic agrochemicals (Gai and Wang, 2024). Conventional plant protection strategies, particularly the excessive application of chemical pesticides, have led to severe ecological impact, including soil and water contamination, loss of beneficial microbiota, and the emergence of resistant pathogen strains (Verma et al., 2024). These challenges demand the urgent need for sustainable, eco-compatible, and adaptive disease management approaches that can support long-term agricultural resilience. Integrated Pest Management (IPM), which combines biological, cultural, and chemical strategies, has emerged as an alternative way to minimise pesticide dependency and delay the development of resistance (Washim et al., 2024). Increasing global food demand, along with the impacts of climate change on pest and pathogen dynamics, requires the exploration of novel biological alternatives that ensure both productivity and environmental sustainability. Recent advancements in molecular biology, synthetic biology, and nanotechnology can lead to the development of next-generation biocontrol agents with high specificity and reduced ecological footprint (Patel et al., 2025).

Among these innovations, antimicrobial peptides have obtained considerable attention as potent, natural, and sustainable alternatives to conventional pesticides (Singh et al., 2026). Generally, AMPs are small, cationic molecules with a charge range of +2 to +9 that electrostatically interact with anionic microbial membranes, resulting in membrane disruption and microbial cell death (Meng et al., 2010). These peptides can be considered as natural organic antibiotics produced by living organisms (Gan et al., 2021; Salas-Ambrosio et al., 2021). In the last few decades, these low-molecular-weight AMPs (≥50 amino acid residues) have shown potential for crop disease and pest management due to their vigorous activity against microorganisms (Lopez-Garcia et al., 2012). Plant disease management using biological control methods is a matter of great concern, and the application of AMPs is currently supported by modern biotechnology as an eco-friendly alternative to synthetic pesticides (Afridi et al., 2022). AMPs also have broader biological functions, including the regulation of immune responses, iron uptake, and cellular growth (Fukuta et al., 2012). It has also been reported that two AMPs can act synergistically to provide greater protection than single-peptide applications in some plant defence systems (Magana et al., 2020; Mendes et al., 2021).

AMPs utilise various strategies to fight plant pathogens, thereby reducing the likelihood of resistance development in pathogens. A primary action mechanism is through pathogen cell membrane disruption (Benfield and Henriques, 2020). The amphipathic nature of AMPs allows them to insert into the bacterial lipid bilayer, leading to pore formation or membrane disruption. This damages the integrity of the membrane and results in cellular contents leakage, osmotic destabilisation, and cell death (Li et al., 2017). In addition to membrane localisation, AMPs can also enter cells and inhibit essential intracellular activities, such as DNA replication, protein production , and cell-wall biosynthesis (Mardirossian et al., 2014). They interfere with primary biosynthetic pathways by binding to vital macromolecules, disrupting metabolic processes and preventing pathogen growth. In addition, biofilms can be destroyed by the insertion of AMPs into the extracellular matrix, thereby preventing microorganism proliferation (Naaz et al., 2023). These mechanisms contribute to the broad-spectrum and efficient antimicrobial action of AMPs in protecting against various plant pathogens. AMPs also support the innate immune defence in plants by acting as immunomodulatory agents, inducing host defence responses, signalling cascades, and stress tolerance pathways that enhance systemic resistance in plants (Kawmudhi et al., 2025; Ioannou et al., 2023). The agricultural promise of AMPs includes controlling plant diseases, improving plant disease resistance, creating disease-resistant crops, and extending the shelf life of agricultural products (Fadiji et al., 2023; Nazarian-Firouzabadi et al., 2024). The plant-derived AMP showed broad-spectrum bactericidal activity against a variety of phytopathogens like *Corynebacterium flaccumfaciens, C. fascians, C. michiganense, C. poinsettiae, C. sepedonicum, Erwinia amylovora, Pseudomonas solanacearum* and *Xanthomonas phaseoli* and *X. campestris* (Caleya et al., 1972; Salas et al., 2015). Several studies have demonstrated in recent years that enhanced expression of AMPs in transgenic plants leads to a significant reduction in pathogen proliferation within host tissues (Deo et al., 2022; Hao et al., 2016; Mirzaee et al., 2021; Zhao et al., 2025). The exogenous application of AMPs has been developed as a practice to control plant diseases, demonstrating the potential of these peptides to serve as new active ingredients against pathogens (Huang et al., 2021).Structurally diverse, encompassing linear, cyclic, and β-sheet peptides, AMPs interact with microbial membranes through electrostatic and hydrophobic interactions, resulting in pore formation and membrane destabilisation (Gan et al., 2021). Several AMPs have been engineered or synthetically modified to enhance stability, target specificity, and field applicability, positioning them as promising bioformulations for sustainable crop protection (Sun et al., 2025). The dual functionality of AMPs, encompassing antimicrobial and immune-modulatory mechanisms, presents a unique opportunity for their deployment as “bioweapons” in sustainable Plant disease management, particularly against resistant plant pathogens.

This review comprehensively studies antimicrobial peptides as next-generation, sustainable alternatives to chemical pesticides for plant disease management. It includes the global challenges caused by increasing plant disease incidence, pesticide overuse, ecological disruption, and pathogen resistance, and positions AMPs as eco-compatible biocontrol agents with broad-spectrum antimicrobial and immunomodulatory properties. The review covers the structural diversity, classification, and molecular mechanisms of plant- and microbe-derived AMPs, with respect to pathogen prevention by membrane disruption, intracellular targeting, and activation of host defence signalling networks, including AMP-associated gene interaction pathways. Understanding the functional dynamics of AMPs and their integration into existing plant protection structures could revolutionise sustainable agriculture by reducing reliance on chemical pesticides and promoting ecological resilience.

## 2 Antimicrobial peptides: an overview

Antimicrobial peptides (AMPs) constitute a diverse group of small, naturally occurring bioactive molecules exhibiting broad-spectrum activity against diverse microorganisms as well as ability to bypass the resistance mechanisms of classical antibiotics (Sahoo et al., 2021). They are integral components of the innate immune defence of all domains of life, including microorganisms, plants, invertebrates, and vertebrates. Advancements in computational biology and bioinformatics have revolutionised the discovery, characterisation, and functional classification of AMPs, enabling their exploitation as next-generation bioweapons for sustainable PDM.

Emerging hierarchical multi-label models, such as HMD-AMP, have enhanced the predictive classification of AMPs by assigning multiple functional labels ranging from antibacterial and antifungal to anti-insect and antiparasitic activities, thereby displaying a multidimensional understanding of AMP versatility (Yu et al., 2021). Similarly, the AMP Cliff framework shows “activity cliff” peptide pairs-sequences with minimal structural variations but markedly different bioactivities- demonstrating the limitations of sequence similarity-based prediction and emphasising the need for multi-parameter functional evaluation (Li et al., 2025). Ensemble learning approaches further refine AMP prediction by integrating physicochemical, evolutionary, and secondary structural attributes to yield robust, high-confidence classifiers (Lertampaiporn et al., 2021). Bayesian network-based models have demonstrated high efficacy (up to 94% accuracy) in detecting sequence motifs and amino acid distributions associated with antimicrobial and multifunctional activity, providing interpretability and mechanistic insights into AMP function (Barrett et al., 2018). These computational frameworks have revolutionised AMP bioinformatics, laying the groundwork for rational peptide design and precision-driven biocontrol applications in agriculture.

AMPs are ubiquitously distributed across biological kingdoms, where they display crucial roles in innate immunity and ecological resilience. In mammals, defensins and cathelicidins act as primary antimicrobial agents, whereas amphibians, such as frogs, secrete magainins with potent bactericidal activity. Insects synthesise peptides, such as cecropins, defensins, and attacins, which serve as vital defences against pathogenic invasion (Dini et al., 2022). Marine invertebrates and crustaceans also deploy structurally diverse AMPs as part of their innate defence systems (Dini et al., 2022).

In plants, AMPs such as thionins, defensins, and cyclotides form an essential component of host defence and are predominantly expressed in seeds, leaves, and roots. These peptides are abundant in prominent agricultural families, such as Solanaceae, Fabaceae, and Brassicaceae, and are also widely found in cereals, including wheat, maize, and barley (Zou et al., 2023a). Their ability to disrupt pathogen cell membranes or interfere with intracellular targets positions them as promising eco-friendly alternatives to chemical pesticides.

Microbial sources such as *Bacillus*, *Streptomyces*, and lactic acid bacteria synthesise bacteriocins and lantibiotics with potent antimicrobial properties, many of which hold translational potential in plant protection and food preservation (Ali et al., 2024; Chen et al., 2025a; Vojnovic et al., 2024). Extremophilic and marine microorganisms have recently emerged as reservoirs of novel AMPs exhibiting strong antimicrobial (Lin et al., 2023). The symbiotic associations, such as those between entomopathogenic nematodes and their bacterial partners *Xenorhabdus* and *Photorhabdus*, are gaining recognition for producing unique AMPs with dual roles in host defence and insect pathogenesis (De Mandal et al., 2021; Sun et al., 2025).

### 2.1 Structural diversity and functional characteristics

AMPs exhibit structural diversity and functional adaptability, making them favourable molecular weapons for sustainable PDM. Their structural classes include linear, cyclic, α-helical, β-sheet, and hybrid peptides, often further stabilised through post-translational modifications such as cyclisation, incorporation of D-amino acids, or lipidation, which enhance their resistance to proteolytic degradation and improve bioactivity under field conditions (Gan et al., 2021). Recent structural innovations, such as AMP–drug conjugates, dendrimers, and peptidomimetics, have expanded their functional landscape, offering opportunities for engineering peptide-based biocontrol agents (Gan et al., 2021).

Protein-fold databases, such as CATH and SCOP, have revealed evolutionary patterns linking distinct structural motifs, including β-barrels and αβ-sandwiches, to specific antimicrobial activities (Alvarez-Carreño, 2025). These correlations between structure and function have been substantiated by experimental studies, which show that α-helical AMPs exhibiting variations in amphiphilicity and helicity govern their ability to disrupt microbial membranes or translocate into intracellular targets. This suggests that peptides with similar secondary structures can exert their effects through diverse mechanisms, including membrane permeabilisation, inhibition of microbial enzymes, interference with quorum sensing, and modulation of host immune responses (Balleza, 2025).

β-sheet and extended AMPs, stabilised by disulfide bonds, display enhanced conformational rigidity and are particularly effective in binding to multiple microbial components such as cell wall precursors or nucleic acids, conferring broad-spectrum defence potential against plant pathogens (Li et al., 2022). The computational and machine learning-based predictions indicate that physicochemical parameters, such as net charge, hydrophobicity, amphipathicity, and molecular flexibility, can be used to classify AMPs into functional subgroups with antibacterial, antifungal, antiviral, or antiparasitic activities (Tan et al., 2021).

### 2.2 Genes associated with antimicrobial peptides and their regulation

**Figure 1:**
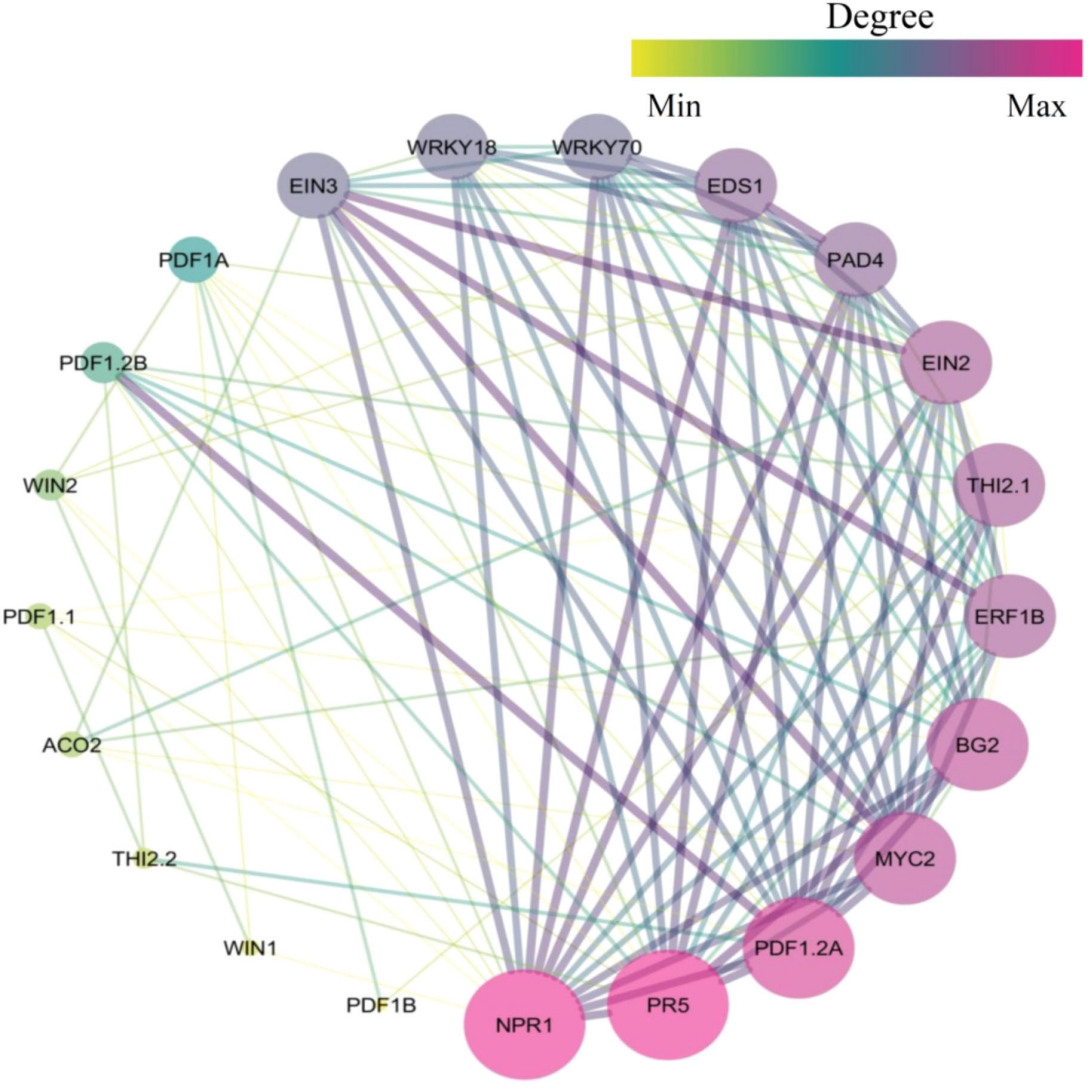
Topological networking of genes associated with antimicrobial peptides using the Cytoscape platform (version 3.10.3; https://cytoscape.org/, accessed on 27 October 2025).

Plants possess an intricate defence network mediated by the production of antimicrobial peptides, which serve as natural antibiotics against invading pathogens (Li et al., 2021). The constructed network of genes involved in antimicrobial peptide production identified 21 genes (**Figure 1**) that exhibit a network density of 0.538, indicating a high level of interconnectivity. The homogeneity value of 0.440 reflects moderate uniformity in node connectivity across the network. An average path length of 1.495 and an average number of neighbours of 10.762 indicate efficient information flow and the presence of tightly connected gene clusters involved in antimicrobial peptide synthesis. The AMPs are small, cationic molecules acting as natural antibiotics against bacterial, fungal, and viral pathogens. Diverse gene families encode these AMPs and are tightly regulated by phytohormonal and transcriptional networks (Jiang et al., 2025). Defensin genes (PDF1.2) are strongly induced during pathogen invasion, primarily through the jasmonic acid and ethylene signalling pathways, under the control of transcription factors such as ERF1, EIN3, and WRKY70 (Kosaka et al., 2020; Li et al., 2004). Thionins (Thi2.1) and LTPs are also upregulated upon pathogen attack through salicylic acid-dependent signalling cascades mediated by NPR1, EDS1, and PAD4, which activate systemic acquired resistance (Edqvist et al., 2018; Ha-Tran and Huang, 2025; Seyfferth and Tsuda, 2014).

Hevein-like peptides (AtHEL) and GASA/Snakins (AtGASA4, StSN1) are transcriptionally modulated by redox-sensitive transcription factors and gibberellin-responsive elements, linking defence signalling to developmental processes (Nahirñak et al., 2012; Slavokhotova et al., 2017; Su et al., 2020). Cyclotides (Kalata B1–B8, ClCYC1) are produced as precursor peptides that undergo post-translational cyclisation in response to stress or wounding stimuli, thereby enhancing their structural stability and antimicrobial potency (Daly et al., 2006; Huang et al., 2019). Pathogenesis-related (PR) genes (PR-1a, PR-5, OsPR10a) are activated through SA- and ET-dependent signalling networks during hypersensitive responses (Anisimova et al., 2021; dos Santos and Franco, 2023).

Regulatory proteins, such as WRKY18, MYC2 (Myelocytomatosis 2), EIN2 (Ethylene Insensitive 2), ACO2 (Aconitase 2), and CTR1 (Copper Transporter 1), coordinate crosstalk between the jasmonic acid, salicylic acid, and ethylene pathways to regulate AMP gene expression (Bolouri Moghaddam et al., 2015; Li et al., 2019). Cyclophilins participate in post-translational modification and stabilisation of AMP proteins, ensuring their functional conformation under stress (Kumari et al., 2013; Singh et al., 2020). These AMP-related and regulatory genes form a defence network, integrating hormonal signalling, transcriptional control, and post-translational regulation. These mechanisms provide fundamental understandings for developing transgenic or genome-edited crops with enhanced resistance to pathogens, supporting sustainable and eco-friendly agricultural systems.

### 2.3 Mechanisms of action of antimicrobial peptides against pathogens

**Figure 2:**
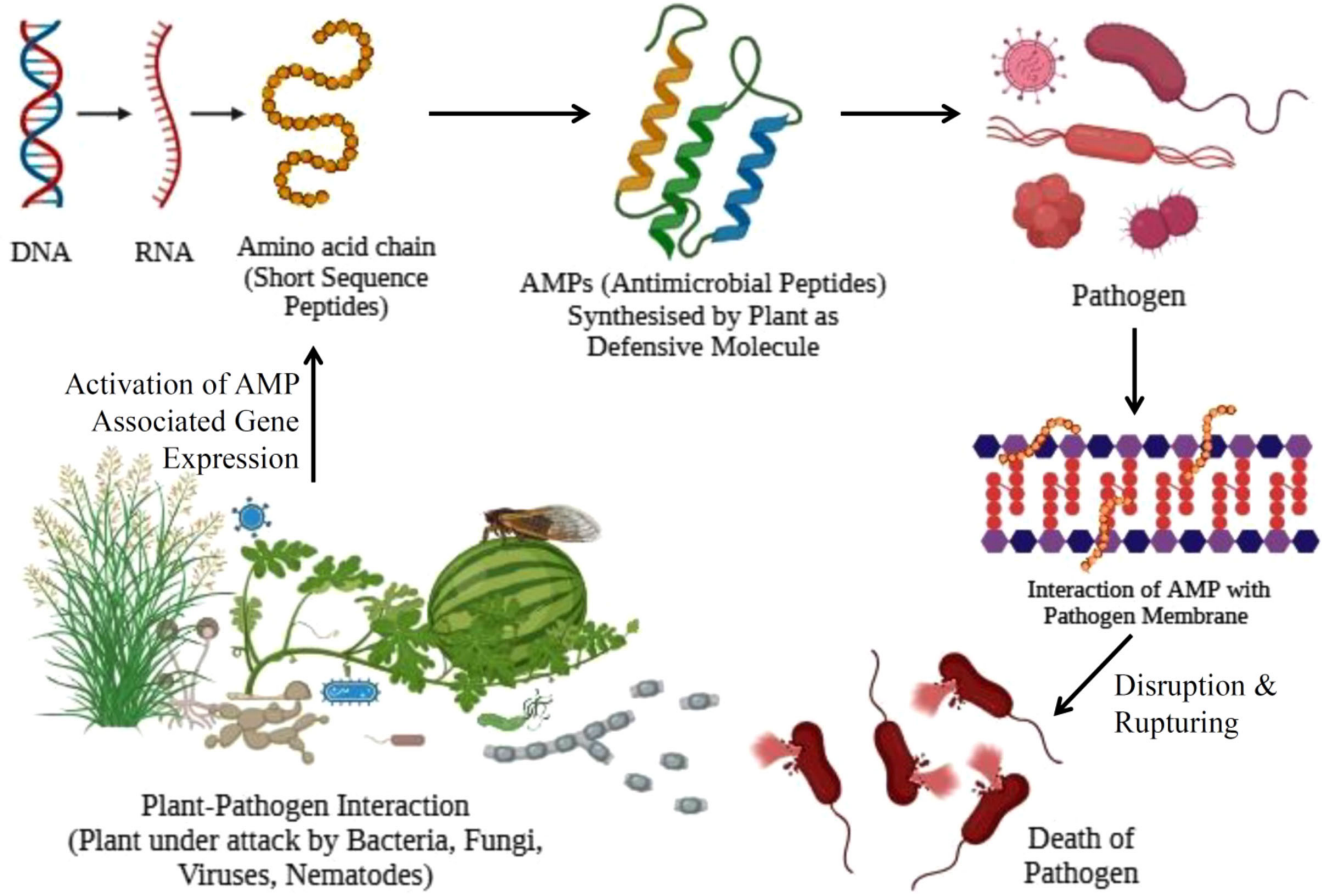
Schematic representation of antimicrobial peptide mediated plant defence. Pathogen attack activates AMP-associated gene expression, leading to the expression of short amino acid sequences that form functional antimicrobial peptides. These AMPs interact with pathogen cell membranes, causing membrane disruption, rupture, and ultimately pathogen death and providing immunity to plants.

AMPs represent a vital component of the innate immune defence in both plants and animals, functioning as adaptable molecules with wide-spectrum activity against pathogenic microorganisms. Their primary mode of action involves direct interaction with microbial membranes, leading to disruption of membrane integrity, pore formation, and eventual cell lysis (Gagandeep et al., 2024) (**Figure 2**). This membranolytic activity is due to electrostatic interactions between the cationic AMPs and the anionic phospholipid components of microbial membranes, thereby inducing rapid pathogen death. AMPs also act as potent modulators of host immunity, as they activate pattern recognition receptors (PRRs), such as Toll-like receptors (TLRs) and receptor-like kinases (RLKs), triggering downstream signalling cascades that enhance local and systemic immune responses (Lee et al., 2019).

AMPs influence epigenetic regulation and chromatin remodelling processes, thereby modulating defence-related gene expression during pathogen attack (Kim, 2019). This transcriptional reprogramming ensures rapid activation of immune responses and contributes to long-term defence priming. Studies have also shown the involvement of AMPs in microRNA-mediated immune memory, facilitating adaptive-like responses that enable plants to respond more effectively upon subsequent pathogen exposure (Luo et al., 2024).

Plant-derived antimicrobial peptides support innate immunity in plants by targeting a range of pathogens, including bacteria, fungi, and viruses (de Oliveira et al., 2024). Their primary mechanism involves electrostatic interactions with negatively charged microbial membranes, leading to membrane insertion, pore formation, membrane destabilisation, and cell lysis. Some AMPs penetrate the cell and inhibit intracellular targets such as DNA, RNA, and essential enzymes, thereby disrupting metabolic processes (Li et al., 2021). In plants, AMPs such as defensins and thionins can initiate ROS production, callose deposition, and the upregulation of PR proteins, collectively strengthening the plant’s defence barriers (Gu et al., 2025).

## 3 Plant-derived AMPs: natural defenders against pathogens

AMPs act as the first line of defence against a broad spectrum of plant pathogens, including bacteria, fungi, and viruses, thereby maintaining plant health and resilience under biotic stress (Li et al., 2021). These peptides exhibit rapid and targeted antimicrobial activity through mechanisms such as membrane permeabilisation, interference with intracellular targets, and modulation of host defence signalling pathways.

The molecular and cellular basis of plant innate immunity is mainly catalysed by antimicrobial peptides (AMPs). When pathogens attack (bacteria, fungi, viruses, or nematodes), plants recognise pathogen-associated molecular patterns (PAMPs) through pattern recognition receptors, thereby triggering defence signalling cascades. This leads to the activation of AMP-associated genes at the transcriptional level (**Figure 2**). The activated genes are transcribed into RNA, which is subsequently translated into short amino acid chains, producing precursor peptides. These peptides become active upon proper folding and, in some cases, upon post-translational modifications, generating functional antimicrobial peptides. Once synthesised, AMPs are targeted to the site of infection, where they directly interact with pathogen cell membranes. Due to electrostatic attraction between the positively charged AMPs and negatively charged microbial membranes, AMPs insert into the lipid bilayer. This interaction causes membrane destabilisation through pore formation, thinning, or complete rupture. Disruption of membrane integrity leads to leakage of cellular contents, loss of membrane potential, and impairment of essential metabolic processes, resulting in pathogen death and thus protecting the plant from infection.

In recent years, growing concerns over antibiotic resistance and the environmental hazards of synthetic pesticides have intensified research on plant-derived AMPs as sustainable and eco-friendly alternatives for crop protection (Sun et al., 2025). Their structural diversity, stability, and multifunctional nature make them promising candidates for the development of next-generation biopesticides and transgenic crops with enhanced disease resistance. Thus, plant-derived AMPs represent an innovative frontier in sustainable PDM, integrating natural defence strategies with modern biotechnological advancements.

### 3.1 Mitogen-activated protein kinase cascades regulating WRKY-Driven AMP expression in plants

**Figure 3:**
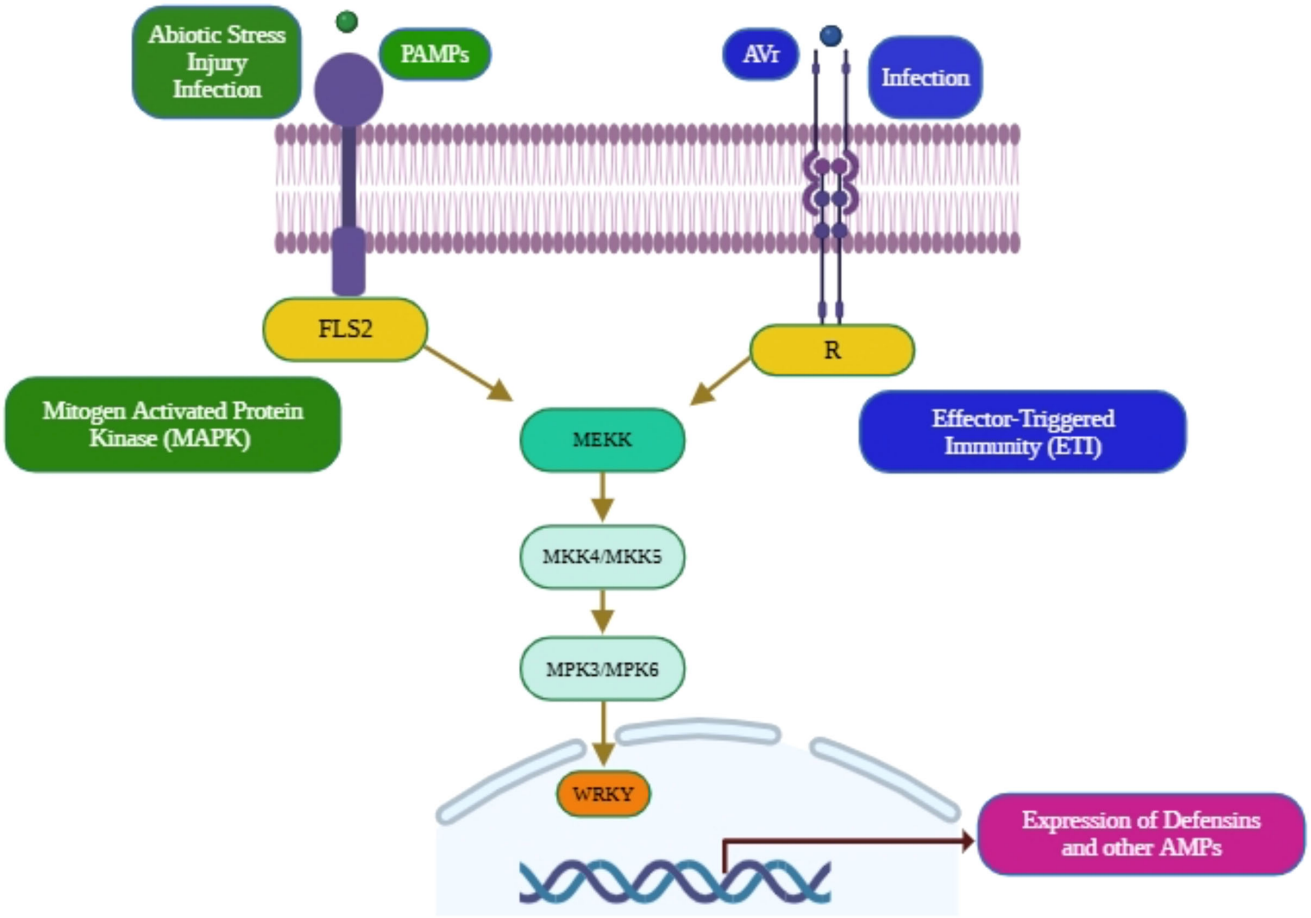
Signalling cascade triggering AMP synthesis in response to pathogen infection.

**Figure 4:**
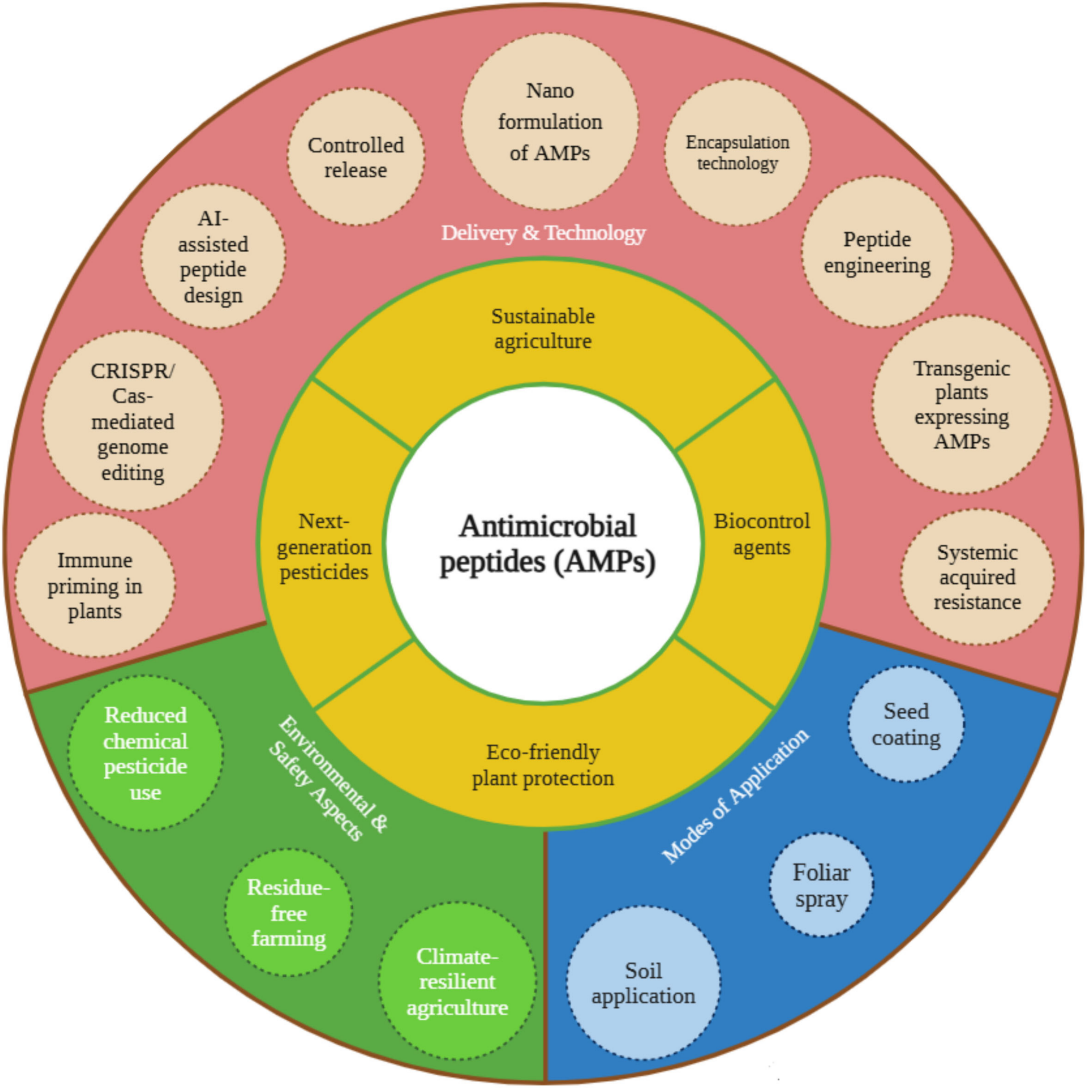
Application of antimicrobial peptides in sustainable agriculture.

The recognition of pathogen-associated molecular patterns (PAMPs) by plant pattern recognition receptors (PRRs) initiates a cascade of immune signalling events leading to the expression of antimicrobial peptides (AMPs), which serve as vital components of plant innate defence (Chen et al., 2025b; Hou et al., 2019; Zhang and Zhou, 2010). Among the well-characterised PRRs, the flagellin-sensing 2 (FLS2) receptor specifically perceives the bacterial PAMP flg22 and, upon activation, forms a complex with the co-receptor BAK1 to trigger downstream signalling (Garcia et al., 2014; Sun et al., 2012). This activation initiates the MAPK cascade, wherein MAPK/ERK kinase kinases (MEKKs), particularly MEKK4 and MEKK5, phosphorylate MAPK kinases (MKK4/5), which in turn activate MPK3 and MPK6 (Jiang et al., 2022). These terminal kinases phosphorylate transcription factors such as WRKYs, which bind to W-box elements in the promoters of AMP-encoding genes, thereby enhancing their transcriptional activation (Bakshi and Oelmüller, 2014). The effector proteins (Avr) secreted by pathogens are recognised by corresponding intracellular resistance (R) proteins, which activate effector-triggered immunity (ETI) that converges on similar MAPK signalling modules, thereby amplifying AMP gene expression and other defence responses (Nimchuk et al., 2001; Petit-Houdenot and Fudal, 2017). Thus, the PAMP–PRR and Avr–R recognition systems integrate through MEKK–MKK–MPK signalling cascades to activate WRKY transcription factors, required for AMP production and effective plant defence against microbial pathogens (Lang and Colcombet, 2020; Devendrakumar et al., 2018) (**Figure 3**). Dummy **Figure 4**, Dummy **Table 1**.

**Table 1:** Mechanisms of antimicrobial action and potential applications of major families of plant antimicrobial peptides.

AMP family	Mechanism of antimicrobial action	Agricultural applications	References
Plant defensins	Binds with fungal membrane sphingolipids, leading to pore formation, membrane permeabilisation, and ion flux disruption. Produces ROS and inhibits fungal growth signalling	Transgenic crops resistant to *Fusarium* sps., *Verticillium* sps., *Botrytis* sps.; seed protection	Thomma et al. (2002)
Thionins	Highly cationic peptides causing membrane disruption and cell lysis	Cereal crop protection	Saberi Riseh et al. (2025)
Hevein-like peptides	Chitin binding and inhibition of fungal cell wall synthesis; membrane destabilisation	Engineering resistance against chitin-containing fungi	Odintsova et al. (2020)
Cyclotides (cystine-knot peptides)	Highly stable cyclic peptides; membrane disruption; resistance to proteolysis	Durable biofungicides; bioinsecticides; peptide engineering scaffold	Craik et al. (1999)
Lipid transfer proteins (LTPs)	Bind membrane lipids, causing membrane permeabilisation; inhibit fungal spore germination	Transgenic fungal resistance; post-harvest protection	Kader (1996)
Snakins (GASA peptides)	Membrane permeabilisation; interaction with metal ions and redox pathways	Resistance to bacterial and fungal pathogens; tuber protection	Segura et al. (1999)
Thaumatin-like proteins (PR-5)	β-glucan binding; membrane permeabilisation; inhibition of hyphal growth	Fungal resistance breeding; stress-responsive marker genes	Feng et al. (2024)
Protease inhibitors (small AMP-like peptides)	Inhibit microbial and insect proteases that reduce virulence and digestion	Insect-resistant transgenic crops; integrated pest management	Ryan (1990)
Glycine-rich peptides	Chitin binding; RNase/DNase-like activity; fungal growth inhibition	Cereal disease resistance improvement	Roy et al. (2023)
Puroindolines	Amphipathic peptides interacting with microbial membranes	Grain defence; resistance to storage fungi	Morris (2002)

### 3.2 Categories of plant AMPs

Due to their structural and functional diversity, plant-derived antimicrobial peptides are categorised into several families, each possessing distinct biochemical features and antimicrobial mechanisms (de Oliveira et al., 2025). These AMPs represent an integral component of the plant innate immune system, acting as natural bioweapons that target a broad spectrum of plant pathogens, including fungi, bacteria, and viruses. Their stability, membrane-targeting ability, and low likelihood of resistance development make them promising eco-friendly alternatives to chemical pesticides in sustainable crop protection.

#### 3.2.1 Defensins

Initially classified as γ-thionins, plant defensins were first discovered in barley (*Hordeum vulgare*) and wheat (*Triticum aestivum*). These small, basic, cysteine-rich peptides (~5 kDa; 45–54 amino acids) exhibit potent antifungal, antibacterial, and enzyme-inhibitory activities (de Oliveira et al., 2025). Structurally, they comprise a triple-stranded β-sheet and an α-helix stabilised by four disulfide bridges, which confer high thermal and proteolytic stability. Their mechanism of action involves interaction with membrane glucosylceramides, leading to membrane destabilisation, ion efflux, and subsequent cell death. Based on their functional diversity, defensins are categorised into two groups: those that induce morphological deformations in fungal hyphae and those that inhibit growth without structural distortion (Nawrot et al., 2014). Evolutionarily, plant defensins share homology with insect and mammalian defensins, indicating a conserved defence lineage.

#### 3.2.2 Thionins

Thionins are low molecular weight (~5 kDa), cationic peptides enriched in cysteine, arginine, and lysine residues. Their tertiary structure comprises two antiparallel α-helices and a double-stranded β-sheet stabilised by multiple disulfide bonds, conferring strong amphipathic character. Thionins interact electrostatically with negatively charged phospholipids in microbial membranes, resulting in pore formation and cytoplasmic leakage (Mazurkiewicz-Pisarek et al., 2023). These peptides exhibit broad-spectrum toxicity against fungi, yeasts, and bacteria, and are widely distributed across both monocots and dicots, with over 100 known variants. Their rapid membrane-disruptive action has positioned them as natural templates for developing peptide-based biofungicides (Katharina et al., 2021).

#### 3.2.3 Cyclotides

Cyclotides are cyclic cystine-knot peptides characterised by a head-to-tail cyclised backbone and three intramolecular disulfide bonds forming a cystine knot motif (Gruber et al., 2007). Comprising 28–37 amino acids, they exhibit exceptional stability against thermal, enzymatic, and chemical degradation. Cyclotides are primarily classified into Möbius and bracelet subfamilies, which differ in the presence of a cis-proline residue (Craik et al., 1999). Identified in families such as Violaceae, Rubiaceae, Cucurbitaceae, and Poaceae, they disrupt microbial membranes through detergent-like activity. Due to their stability and bioactivity, cyclotides are emerging as promising scaffolds for engineering synthetic AMPs with enhanced agronomic potential (Poth et al., 2011).

#### 3.2.4 Snakins

Snakins, originally isolated from potato (*Solanum tuberosum*) tubers, are cysteine-rich peptides (~6.9 kDa) composed of 63 amino acids with 12 conserved cysteines forming six disulfide bridges. Snakin-1 (StSN1) and Snakin-2 (StSN2) exhibit broad-spectrum antimicrobial activity against bacterial and fungal plant pathogens. While StSN1 is constitutively expressed in various tissues, StSN2 expression is induced upon pathogen invasion or wounding (Su et al., 2020). Although their precise mechanism remains unclear, their strong cationic nature suggests involvement in membrane interactions or signalling pathways associated with plant defence.

#### 3.2.5 Hevein-like peptides

Hevein, a 4.7 kDa chitin-binding peptide first identified in the latex of the rubber tree (*Hevea brasiliensis*), represents the prototype of hevein-like AMPs. These peptides recognise and bind chitin in fungal cell walls, thereby inhibiting hyphal growth. Structurally, they comprise a conserved chitin-binding domain (20–40 amino acids) rich in cysteine and glycine residues (Loo et al., 2021). Variations exist in the number of disulfide bonds among plant species, ranging from three to five, which contribute to structural diversity and functional adaptability. The presence of hevein-like AMPs in diverse taxa, such as *Avena sativa*, *Ginkgo biloba*, and *Triticum kiharae*, indicates their evolutionary significance in chitin-targeted defence mechanisms (Wong et al., 2016).

#### 3.2.6 Non-specific lipid transfer proteins

Non-specific lipid transfer proteins are ubiquitous in higher plants and display dual roles in physiological processes and defence against pathogens. These proteins consist of four α-helices enclosing a hydrophobic cavity, stabilised by four disulfide bonds (McLaughlin and Tumer, 2025). This cavity accommodates various lipid molecules, facilitating membrane repair and signalling in response to pathogen attack. They can insert into fungal membranes, forming pores that lead to ion efflux and cell death. They are grouped into LTP1 (~9 kDa) and LTP2 (~7 kDa) subfamilies, with conserved glycine and proline residues across monocots and dicots. They participate in cutin biosynthesis, stress adaptation, and antifungal defence in plant immunity (Vriens et al., 2014).

### 3.3 Role in innate immunity and plant defence

Antimicrobial peptides act as rapid and potent bioweapons against invading pathogens. When a pathogen infects and is recognised by pathogen-associated molecular patterns (PAMPs) by pattern recognition receptors, plants activate complex signalling cascades that induce the synthesis and mobilisation of AMPs to infection sites (Islam et al., 2024). These peptides exert their defensive functions either directly by targeting and killing pathogens or indirectly by modulating immune signalling pathways to enhance host resistance.

Plant-derived AMPs exhibit direct antimicrobial activity by interacting with microbial cell membranes, resulting in membrane permeabilisation, leakage of cellular contents, and ultimately, cell death (Zhang et al., 2021). Their cationic and amphipathic nature enables them to bind specifically to the negatively charged components of bacterial and fungal membranes, such as phospholipids, lipopolysaccharides, or lipoteichoic acids (van der Weerden & Anderson, 2013). After attachment, AMPs can destabilise membrane integrity through mechanisms prescribed by the barrel-stave, toroidal-pore, or carpet models, each culminating in membrane thinning, pore formation, and cytoplasmic leakage (Zhang et al., 2021). Like the radish defensin Rs-AFP2, which interacts with fungal glucosylceramides in Fusarium culmorum, leading to membrane permeabilisation and ion flux disruption (Luo et al., 2021).

Beyond membrane disruption, certain AMPs exhibit intracellular activities by penetrating microbial cells and targeting essential metabolic processes, including DNA replication, RNA transcription, and protein synthesis (Gagandeep et al., 2024). Some thionins and defensins interfere with ribosomal function or induce oxidative stress, further impairing pathogen viability.

AMPs also function as critical regulators of plant immunity; they can act as signalling molecules that activate defence-related pathways, such as the salicylic acid and jasmonic acid pathways, leading to the induction of PR genes and the activation of systemic acquired resistance (Bolouri Moghaddam et al., 2015). For instance, the plant defensin PDF1.2, whose expression is upregulated through JA-dependent signalling, shows enhanced resistance against necrotrophic fungal pathogens.

## 4 AMPs from microbial sources for plant protection

Antimicrobial peptides derived from bacteria and fungi have emerged as potent natural bioweapons for sustainable PDM due to their structural diversity, broad-spectrum antimicrobial properties, and environmental safety. Among microbial AMPs, bacteriocins and lipopeptides have gained substantial attention for their efficacy against plant pathogens and their potential role as eco-friendly alternatives to synthetic pesticides.

Bacteriocins, a diverse group of ribosomally synthesised AMPs produced by bacteria, exhibit potent inhibitory activity against a range of plant pathogenic microbes through mechanisms such as pore formation, cell wall disruption, and inhibition of nucleic acid synthesis (Iralu et al., 2024). Their narrow-spectrum antibiotics are often targeted against Gram-positive pathogens, thereby decreasing collateral effects on beneficial microbiota and preserving soil and rhizosphere health. Lantibiotics, a subclass of bacteriocins, have demonstrated significant inhibitory activity against soil-borne fungal pathogens, such as *Fusarium* and *Rhizoctonia* spp., offering a sustainable strategy for controlling wilt and rot diseases. The stability, specificity, and low potential for resistance development of bacteriocins make them ideal candidates for incorporation into integrated pest management systems.

In addition to bacteriocins, lipopeptides secreted by soil-associated bacterial genera such as *Bacillus* and *Pseudomonas* constitute another critical class of microbially derived AMPs with pronounced antifungal and anti-biofilm properties (De Mandal et al., 2021). These amphiphilic molecules, exemplified by surfactins, fengycins, and iturins, exert their antifungal action primarily by disrupting the fungal cell membrane, leading to cytoplasmic leakage and cell death. Their ability to inhibit spore germination and hyphal growth in pathogenic fungi such as *Fusarium*, *Verticillium*, and *Pythium* enhances their utility as biocontrol agents in crop protection. Moreover, certain lipopeptides can form metal ion complexes and maintain activity under abiotic stress conditions such as salinity and temperature fluctuations, making them robust candidates for field applications under variable environmental conditions.

Microbial-derived AMPs deliver a green, biodegradable, and target-specific solution for plant protection. Their low toxicity to humans, animals, and non-target organisms, coupled with their biodegradability, positions them as sustainable alternatives to conventional agrochemicals. Advances in genetic engineering and synthetic biology have opened new avenues for enhancing AMP production, including the development of transgenic plants expressing AMP genes or bioinoculants engineered to secrete antimicrobial peptides in the rhizosphere (Iralu et al., 2024).

## 5 Synergistic interactions with plant immunity

The synergistic role of antimicrobial peptides in modulating plant immunity has garnered significant attention due to their dual function in both direct pathogen inhibition and immune regulation. AMPs act as essential modulators of innate plant defence signalling. Recent evidence suggests that AMPs can activate multiple defence-related signalling cascades, including MAPKs, reactive oxygen species (ROS) generation, and phytohormonal crosstalk, which are crucial for both PAMP-triggered immunity (PTI) and effector-triggered immunity (ETI) (Li et al., 2021). This activation leads to enhanced and sustained defence response against a broad spectrum of plant pathogens.

AMPs exhibit synergistic interactions with endogenous defence molecules and microbial antagonists, thereby amplifying overall disease resistance. For instance, the sweet potato defensin SPD1 not only exerts potent antifungal effects but also modulates cellular redox homeostasis, linking its antimicrobial function to immune priming mechanisms in plants (Mhlongo et al., 2023). Structural studies further reveal that AMP combinations such as VG16KRKP and KYE28 form dimeric conformations that enhance membrane permeability and antimicrobial potency, highlighting the importance of structural dynamics in immune activation.

Beneficial plant-associated microbes, including rhizobacteria and mycorrhizal fungi, can induce AMP expression and potentiate immune responses through microbe-associated molecular pattern (MAMP) signalling, thereby displaying the ecological integration of AMPs within the plant microbiome (Lü et al., 2022). The cooperative interactions between cell-surface pattern-recognition receptors (PRRs) and intracellular nucleotide-binding leucine-rich repeat receptors (NLRs) indicate that AMP-mediated signalling bridges extracellular and intracellular immune networks.

## 6 Biotechnological advances in harnessing antimicrobial peptides for sustainable plant disease management

Microbial-derived antimicrobial peptides have emerged as effective and eco-friendly bio-tools in modern crop protection owing to their biodegradability, target specificity, and minimal environmental footprint. As sustainable alternatives to conventional agrochemicals, AMPs form the cornerstone of next-generation agricultural biotechnology, offering novel strategies to manage a broad spectrum of plant pathogens, including bacteria, fungi, and viruses (Lin et al., 2023).

Advances in genetic engineering, synthetic biology, and genome editing have enabled the transgenic expression of AMP-coding genes in plants, effectively transforming crops into biofactories for AMP biosynthesis. This approach enhances innate plant immunity, providing durable, broad-spectrum resistance against plant pathogens while reducing dependence on chemical pesticides (Sinha and Shukla, 2019). Tools such as CRISPR/Cas-based editing and recombinant DNA technology enable the precise engineering of plant genomes to express AMPs either constitutively or in response to pathogen attack, ensuring efficient and controlled defence responses.

The multifunctional mechanisms of AMPs, including the disruption of microbial membranes, inhibition of protein or nucleic acid synthesis, and interference with intracellular signalling, make them highly effective in curbing pathogen proliferation and preventing the development of resistance, a significant limitation of conventional chemical pesticides (Yao et al., 2023).

Symbiotic bacteria associated with entomopathogenic nematodes have emerged as novel biological sources of AMPs, acting as dual-function biocontrol agents that integrate nematode-mediated pest suppression with peptide-based pathogen inhibition (De Mandal et al., 2021). Field trials have demonstrated that microbial AMP formulations exhibit high efficacy and low phytotoxicity against pathogens such as *Erwinia amylovora*, the causative agent of fire blight, indicating their potential for commercial biocontrol applications (Sabri et al., 2022).

Semi-synthetic and peptide engineering approaches are being employed to optimise AMP stability, specificity, and delivery efficiency under field conditions, thereby expanding their applicability in sustainable crop protection systems (Yang et al., 2025). These biotechnological innovations highlight the transformative potential of AMPs as emerging bioweapons in sustainable PDM, paving the way for an era of eco-intelligent and resilient agriculture driven by molecular precision and biological sustainability.

### 6.1 Genetic engineering strategies: transgenic plants expressing AMPs

The strategic integration of AMP genes into plant genomes through genetic engineering represents a promising approach to reinforce plant immunity and achieve sustainable disease management. Transgenic expression of AMPs has shown significant protection against a broad spectrum of bacterial, fungal, and viral plant pathogens (Ha-Tran and Huang, 2025). These peptides act by disrupting pathogen cell membranes, interfering with microbial metabolism, and activating innate immune responses, thereby functioning as molecular bioweapons within plants (Farvardin et al., 2024).

Several studies have validated the efficacy of AMP-mediated defence in diverse crops. For instance, tobacco plants transformed with a synthetic cecropin B gene exhibited enhanced resistance against *Pseudomonas syringae* (Hightower et al., 1994). While rice plants expressing a thionin-defensin fusion gene displayed strong resistance to *Xanthomonas oryzae* pv. *oryzae*, the causal agent of bacterial blight (Jiang et al., 2020). To optimise the expression and specificity of AMP genes, plant-specific and pathogen-inducible promoters have been developed to minimise off-target and pleiotropic effects.

Advancements such as chloroplast transformation offer compartmentalised AMP overexpression, reducing cytotoxicity and enhancing protein yield (Narra et al., 2025). The integration of precision genome-editing platforms, such as CRISPR/Cas systems, enables targeted AMP gene insertion, thereby mitigating transgene silencing and off-target mutagenesis (Lou et al., 2025). Gene-stacking approaches that combine multiple AMP genes are also being explored to establish broad-spectrum, durable resistance and reduce pathogen adaptability (Han et al., 2024).

Despite these advances, challenges remain concerning biosafety, ecological implications, and public perception of AMP-expressing transgenic crops. Finding solutions against these limitations is crucial for the effective utilisation of AMPs as sustainable biocontrol agents in future crop protection systems (Saurabh, 2026). The genetic engineering of plants for the production of antimicrobial peptides represents a promising approach to sustainable PDM. This strategy indicates the inherent advantages of plant-based expression systems, including scalability, cost-effectiveness, and biosafety for producing AMPs in agricultural and biomedical applications (Tang et al., 2023).

*Nicotiana benthamiana* has emerged as a model host for transient AMP expression due to its high amenability to *Agrobacterium*-mediated transformation and rapid biomass accumulation. Several studies have reported efficient AMP production in *N. benthamiana* using molecular farming approaches. Researchers also reported the expression of elastin-like polypeptide (ELP)-conjugated AMPs, which show high yields and potent antimicrobial activity against *Staphylococcus epidermidis* (Atefyekta et al., 2019). The study also indicated that the peptide’s net charge significantly influences expression efficiency and antimicrobial efficacy. Chaudhary et al. (2024) utilised a synthetic biology-based strategy that integrated molecular virology and analytical chemistry to produce AMPs in *N. benthamiana*, generating yields of up to 2.9 mg per 20 g of leaf tissue (Chaudhary et al., 2024). This improvement, by the development through apoplast-targeted expression, enhanced purification efficiency and scalability. A related study using a tobacco mosaic virus (TMV)-based vector system successfully expressed a *de novo* designed AMP with vigorous dual activity against both plant and human pathogens, generating approximately 0.025 mg/g of infected leaf tissue.

Stable transformation of staple crops provides an ecologically viable route for the continuous expression of AMP. Bundó et al. (2014) showed that the stable accumulation of cecropin A within the rice seed endosperm, where compartmentalisation within protein bodies enhanced peptide stability and downstream recovery (Bundó et al., 2014). Montesinos et al. (2021) further examined BP100 derivatives in rice, revealing that peptide design is critical in minimising phytotoxicity while maintaining vigorous antimicrobial activity (Nadal et al., 2012).

Transgenic expression of AMPs has been shown to enhance plant immunity against diverse plant pathogens without compromising growth or yield. Early research demonstrated that *Xenopus laevis*-derived magainin, when expressed in *Nicotiana tabacum*, showed resistance to *Pseudomonas syringae* pv. *tabaci* (Sun et al., 2025). The alfAFP gene from *Medicago sativa* enhanced resistance to *Verticillium dahliae* and *Alternaria solani* in transgenic potato (*Solanum tuberosum*) plants (Gao et al., 2000).

The radish defensin (Rs-AFP2) expressed in rice imparted broad-spectrum resistance to bacterial blight (*Xanthomonas oryzae* pv. *oryzae*) and sheath blight (*Rhizoctonia solani*) without yield penalties, emphasising the agronomic feasibility of AMP-based protection (Jha and Chattoo, 2010). The cecropin B analogue MSI-99, expressed in rice plants, showed substantial resistance to bacterial leaf blight and blast disease with minimal morphological changes (Sharma et al., 2000; Todaro et al., 2023). In tobacco, the fungal-derived KP4 killer protein from *Ustilago maydis* conferred resistance against *Fusarium* and *Alternaria* spp., illustrating the potential for cross-kingdom peptide utilisation (Vicente et al., 2022).

In legumes, the designed peptide D4E1, expressed in *Medicago sativa*, enhanced tolerance to *Phoma medicaginis* without adversely affecting nitrogen fixation or plant vigour (Castell-Miller et al., 2007). The expression of a hevein-like peptide from *Hevea brasiliensis* in wheat (Triticum aestivum) provided stable and heritable resistance against powdery mildew caused by *Blumeria graminis* f. sp. *Tritici* (Zou et al., 2023b). In maize (*Zea mays*), particle bombardment-mediated expression of the synthetic AMP D2A21 enhanced resistance to *Setosphaeria turcica*, the causal agent of northern corn leaf blight (Shi et al., 2018).

### 6.2 CRISPR/Cas-mediated regulation of antimicrobial peptides for enhanced plant immunity

The CRISPR/Cas genome-editing system has emerged as a significant tool to enhance AMP gene networks in plants, enabling targeted improvement of innate immunity against diverse plant pathogens (Ansori et al., 2023). Through allowing sequence-specific insertion, deletion, or activation of AMP-related genes without introducing foreign DNA, CRISPR/Cas circumvents many of the biosafety and regulatory constraints associated with conventional transgenic approaches (Mhlongo et al., 2023).

A particularly promising strategy involves engineering the promoter regions of endogenous AMP genes to achieve inducible or pathogen-responsive expression. CRISPR activation systems (CRISPRa), which employ catalytically inactive Cas9 (dCas9) fused with transcriptional activators such as VP64 or VPR, have successfully triggered the overexpression of plant defensins and thionins in *Arabidopsis* and tomato, thereby conferring enhanced resistance to bacterial and fungal pathogens (Xu et al., 2023). Conversely, CRISPR interference (CRISPRi), utilising dCas9 coupled with transcriptional repressors (KRAB), has been applied to silence negative regulators of AMP biosynthesis, indirectly stimulating innate defence responses (Asif et al., 2024).

CRISPR/Cas-mediated homology-directed repair (HDR) for precise insertion of synthetic or optimised AMP genes into genomic “safe harbors,” ensuring stable expression while preserving essential native functions. The next-generation derivatives of CRISPR include base and prime editing, enabling fine-tuned point mutations or promoter modifications that enhance peptide stability, antimicrobial potency, and inducibility under stress conditions without generating double-strand breaks (Liao et al., 2024).

Multiplex CRISPR approaches further permit simultaneous modification of multiple AMP-related targets, offering synergistic defence against a broad range of pathogens. The integration of tissue-specific or inducible Cas9 expression systems has improved the spatial and temporal control of AMP regulation, thereby minimising off-target effects and growth penalties (Xu et al., 2023). Despite these advances, challenges such as off-target mutations, delivery efficiency, and regulatory approval of edited crops require further study. Coupling CRISPR/Cas technology with multi-omics platforms, including transcriptomics, proteomics, and metabolomics, will enable the systematic dissection and optimisation of AMP regulatory networks. This strategy promises to accelerate the development of resilient, disease-resistant crop varieties and advance antimicrobial peptides as sustainable alternatives for eco-friendly PDM (Borah et al., 2024).

## 7 Application of antimicrobial peptides in agriculture

Antimicrobial peptides are promising eco-compatible alternatives to synthetic pesticides for sustainable PDM, due to their rapid mode of action, broad-spectrum antimicrobial activity, and minimal risk of resistance development (Kawmudhi et al., 2025). Their multifunctional nature allows effective suppression of plant pathogenic bacteria, fungi, and viruses, offering an efficient and sustainable approach to crop protection. Recent technological innovations have significantly enhanced the agricultural applicability of AMPs. The development of nano-enabled AMP formulations has enhanced peptide stability, bioavailability, and targeted delivery under various environmental conditions (Bhanuprakash et al., 2023). Such nanoformulations ensure sustained peptide release and protection from degradation caused by UV radiation, pH fluctuations, or enzymatic hydrolysis, making them suitable for field application.

Another promising approach involves the genetic engineering of crops to endogenously express. AMPs endogenously, providing continuous protection against invading pathogens without the need for repeated external applications (Tang et al., 2023). Transgenic crops, such as rice, wheat, tomato, potato, banana, and soybean, expressing AMP genes have demonstrated enhanced resistance to both biotic and abiotic stresses, thereby minimising reliance on chemical pesticides (Han et al., 2025). However, commercialisation remains a challenging factor due to inconsistent transgene expression, peptide degradation by endogenous proteases, and biosafety concerns under diverse agroecological conditions.

The integration of bioinformatics and machine learning tools, such as the AGRAMP database, has revolutionised the discovery of AMPs. Computational prediction and screening platforms accelerate the identification of novel AMPs with optimised structural and functional attributes against specific plant pathogens, thereby reducing experimental costs and time (Shao et al., 2024).

Microbial-derived AMPs are also gaining prominence as biocontrol agents due to their selectivity, environmental safety, and biodegradability (Lin et al., 2023). For effective agricultural use, AMP delivery methods, such as foliar spray, root absorption, and seed coating, are being optimised for environmental stability and efficient plant uptake. Synergistic formulations combining AMPs with chemical pesticides or microbial antagonists have demonstrated improved efficacy and delayed pathogen resistance development, thereby enhancing integrated pest management (IPM) programs (Tang et al., 2023).

Exogenous application of AMPs has shown considerable success in post-harvest disease management. Peptides such as PAF56 and the cecropin A-melittin hybrid BP21 effectively control green mould, blue mould, and sour rot in citrus fruits (Wang et al., 2018). Similarly, the synthetic peptide BP15 has been employed in field trials to manage brown spot disease in pears, resulting in a 42%–60% reduction in infection rates (Puig et al., 2015). Food-grade AMPs like ϵ-poly-L-lysine (ϵ-PL) have been successfully utilised against grey mould in tomatoes, representing a safe and environmentally friendly alternative to conventional chemical fungicides (Lv et al., 2022).

### 7.1 Direct use of antimicrobial peptides as biopesticides

AMPs have emerged as promising natural biopesticides due to their broad-spectrum antimicrobial activity, rapid mode of action, and low propensity for resistance development, placing them as suitable candidates for direct application in modern crop protection strategies. Recent advances have demonstrated the feasibility of applying recombinant or chemically synthesised AMPs directly as seed coatings, foliar sprays, or formulations combined with other biocontrol agents. AMPs, such as nisin, defensins, and cecropins, have shown potent activity against a wide range of plant pathogens, including bacteria, fungi, and viruses, as well as certain insect pests (Sun et al., 2025).

The dual utility of AMPs, both in direct external application and transgenic expression, has been substantiated in studies involving peptides derived from plants, bacteria, and fungi. These peptides demonstrate high environmental stability, target specificity, and compatibility with integrated pest management (IPM) systems, thereby reinforcing their suitability for sustainable agriculture (Iralu et al., 2024). The application of recombinant AMPs in combination with monoterpenes for controlling fire blight in pear trees (*Erwinia amylovora*) resulted in a 65% reduction in disease incidence following AMP treatment, confirming the potential of peptide-based formulations as practical biopesticides (Sabri et al., 2022).

### 7.2 Application of AMPs through seed coatings

AMPs have emerged as promising bioactive agents in seed coating technologies, leading to sustainable PDM. Integrating AMPs into biopolymer-based coatings represents a novel approach to strengthening seeds against plant pathogenic invasion, along with enhancing plant vigour. For instance, the incorporation of nisin into silk fibroin-based biopolymer coatings has demonstrated potent antimicrobial efficacy, improving seed germination, seedling growth, and root elongation under abiotic stress conditions, such as cold stress (Jin et al., 2024).

Electrospun polymeric nanofibers have gained attention as carriers for AMPs in seed treatment formulations. While traditionally employed for encapsulating synthetic molecules, electrospinning provides a highly versatile and tunable platform for the controlled and sustained release of AMPs, thereby enhancing their interaction with plant cells and conferring prolonged protection against pathogenic infections (Colín-Orozco et al., 2024).

Microbial-based seed coating systems capable of releasing AMPs or AMP-producing microorganisms show a sustainable route for crop protection. Bio-priming and encapsulation techniques have successfully delivered such coatings, resulting in improved seed germination, enhanced seedling vigour, and reduced dependence on chemical pesticides (Paravar et al., 2023). Studies involving beneficial microbes, such as *Trichoderma guizhouense*, suggest that coating seeds can reshape the rhizosphere microbiome, promoting disease suppression and enhancing soil enzymatic activities, which can be achieved with AMP-based formulations (Xie et al., 2023).

Microencapsulation technologies have been developed to protect bioactive agents under adverse environmental conditions, such as high soil salinity, by ensuring targeted and slow release of active compounds. These properties align well with the requirements of AMP-based delivery systems, ensuring their stability, bioavailability, and sustained biocontrol efficacy in variable field conditions (Sun et al., 2025).

### 7.3 Use of AMPs in combination with other biocontrol agents

AMPs have emerged as potent biocontrol molecules that, when strategically combined with other biological agents, can significantly enhance the efficacy of PDM. Synergistic interactions between plant-derived AMPs, such as 2S albumins and thionins, have demonstrated significant antifungal activity, reportedly up to 73-fold higher inhibition of plant pathogens compared to the use of individual peptides (Taheri-Araghi, 2024). *Bacillus* species serve as prolific producers of diverse AMPs, including surfactins, fengycins, and iturins, which not only exert broad-spectrum antimicrobial activity but also prime plant immune responses through induced systemic resistance. When coupled with *Bacillus*’ inherent biocontrol mechanisms, such as rhizosphere colonisation, competitive nutrient utilisation, and biofilm formation, these AMPs contribute to a multilayered defence system that reinforces plant resilience against multiple pathogens (Huan et al., 2020).

AMP mixtures offer improved suppression of plant pathogenic bacteria while minimising phytotoxicity and delaying the development of resistance (Kawmudhi et al., 2025). This combinatorial approach aligns with the principles of sustainable agriculture by reducing reliance on chemical pesticides and promoting ecologically compatible plant protection.

Advances in genome mining and synthetic biology have accelerated the discovery of novel AMPs from *Bacillus* genomes and other microbial taxa, enabling their incorporation into engineered biocontrol consortia. For instance, genetically modified strains such as *Bacillus subtilis* BBG100, which overproduce the lipopeptide mycosubtilin, display enhanced antagonistic potential against plant pathogens (Leclère et al., 2005). Overexpression of AMPs in microbial platforms provides a scalable, cost-effective, and environmentally sustainable strategy to enhance plant protection and strengthen crop immunity (Jiang et al., 2025).

## 8 Challenges and limitations in the stability and delivery of AMPs

Despite their effective antimicrobial potential, the widespread application of AMPs in sustainable PDM faces several challenges, particularly in terms of bioavailability and delivery efficiency. AMPs are highly susceptible to enzymatic degradation by host and microbial proteases, leading to rapid inactivation under physiological or environmental conditions (Asif et al., 2024). In an agricultural system, exposure to UV radiation, fluctuating pH, and soil-associated enzymes limits their persistence and efficacy.

Structural modifications, such as amino acid substitution, peptide cyclisation, PEGylation, or conjugation with stabilising ligands, have been employed to enhance proteolytic resistance and improve pharmacokinetic stability (Todaro et al., 2023). Such modifications require careful optimisation to retain biological activity while minimising production costs and maintaining ecological safety.

Nanotechnology-based delivery systems have emerged as promising solutions to overcome AMP instability and targeted delivery issues. Encapsulation within lipid nanoparticles, polymeric matrices, metallic nanostructures, and nanogels provides physical protection against degradation and enables sustained or stimulus-responsive release (Min et al., 2024). In relation to plant protection, nanocarrier-mediated delivery of AMPs could enable foliar adherence, enhanced cuticular penetration, and controlled rhizospheric release, thereby improving field-level efficiency and reducing environmental losses. The scalability, formulation complexity, potential phytotoxicity, and environmental risks associated with nanoparticle residues pose new challenges (Islam, 2025).

AMPs often exhibit limited permeability across plant tissues and have a short residence time at infection sites. Advanced biomaterial-based formulations, including hydrogels, electrospun fibres, and bioresponsive carriers, are being developed to ensure prolonged retention, site-specific action, and compatibility with both biotic and abiotic stress conditions (Patrulea et al., 2020). The potential cytotoxicity and immunogenicity of some AMPs may affect the plant-associated microbial community, thereby disrupting the ecological balance of the phytomicrobiome (Jiang et al., 2025). Bacterial resistance mechanisms, such as protease secretion, efflux pump activation, and membrane remodelling, can compromise AMP efficacy over time (Tajer et al., 2024).

The high cost of peptide synthesis, lack of standardised design and testing platforms, and regulatory constraints put limitations on the commercialisation of AMP-based biocontrol formulations. Even synergistic combinations with conventional pesticides or antibiotics require precise formulation to avoid antagonistic effects and preserve biocompatibility (Roque-Borda et al., 2025).

## 9 Commercialised AMPs in agricultural disease management

Antimicrobial peptides originating from diverse natural sources are increasingly recognised as effective and sustainable for plant disease control, offering eco-friendly alternatives to conventional chemical pesticides. Nisin, produced by *Lactococcus lactis*, inhibits bacterial cell wall synthesis and has already been commercialised as a biocontrol agent to manage bacterial pathogens, including *Xylella fastidiosa* subsp. *pauca*, the causative agent of fire blight (Sabri et al., 2024). Dermaseptin, derived from *Phyllomedusa* species, exerts antimicrobial activity through membrane disruption and is currently being investigated for the development of a biopesticide against bacterial wilt disease (Hazime et al., 2022). Similarly, Cecropin B, originally isolated from insects, damages bacterial membranes and is being used for incorporation into transgenic plants to enhance resistance to bacterial leaf blight (Duffield et al., 2023). Thanatin, obtained from the predatory insect *Podisus maculiventris*, also targets microbial membranes and has shown promise as an agricultural biocontrol agent against bacterial spot disease (Dash and Bhattacharjya, 2021). Plant-derived peptides such as Puroindoline from wheat exhibit strong antifungal properties by compromising fungal membrane integrity and demonstrate potential for controlling *Fusarium* head blight (Hu et al., 2022). Beneficial biological control agents and plant growth-promoting microorganisms (BCA-PGPMs), including those associated with amphibian skin (*Xenopus laevis*), contribute to pathogen suppression through nutrient competition and environmental modification, thereby enhancing sustainable crop protection and productivity. Mastoparan, isolated from wasp venom, disrupts membranes and is under evaluation for managing viral plant diseases (Tyśkiewicz et al., 2022). The synthetic peptide BP100 shows broad-spectrum antibacterial and antifungal activity through membrane permeabilisation and is considered a promising preventive treatment for crop protection, particularly in tomato cultivation (Montesinos et al., 2021). These AMPs act by disrupting pathogen membranes, inhibiting cell wall synthesis, or preventing pathogen establishment through competitive interactions.

## 10 Limitations of antimicrobial peptides in plant disease management and future prospects

Antimicrobial peptides have become a sustainable alternative to traditional pesticides. A number of intrinsic restrictions prevent their widespread use in the control of plant diseases (Alzain et al., 2025; Amen et al., 2025; del Olmo and Andreu, 2025). These include the need to improve fermentation and transformation methods, the high costs of synthesis and purification, and the creation of AMPs that are effective against a wider variety of plant diseases, including viruses. The high cost of production is one of the limitations, particularly for chemically synthesised or recombinant peptides, thereby preventing their economic feasibility for agricultural use (Bommarius et al., 2010; Chaudhary et al., 2023; Saubenova et al., 2025). AMPs may exhibit dose-dependent phytotoxicity, where elevated concentrations can disrupt plant cell membranes, alter ion homeostasis, and induce oxidative stress, leading to growth inhibition or tissue injury (Sun et al., 2025). Besides this, AMPs high molecular weight may decrease their ability to prevent infections in plants, and most AMPs show limited penetration across the plant cuticle and cell wall, reducing their systemic movement and effectiveness against internal pathogens (Tang et al., 2023). Repeated applications are required because of their low residual activity, which further escalates the overall cost of disease management (Ali et al., 2025; Kim et al., 2025). For AMPs to be used on a large scale, manufacturing must be extremely economical. AMPs can be produced at a low cost using solid-phase synthesis (Topman et al., 2018), bacterial expression (Schreiber et al., 2017), or eukaryotic expression (Holásková et al., 2018). Some AMPs used in the food and cattle industries, including polylysine, are prepared for plant protection. Even though AMPs are effective against a variety of phytopathogens under laboratory and greenhouse experiments, further field testing under a range of environmental gradients is still anticipated. Improving AMP formulations with greater stability and absorption effectiveness is essential for field use. Besides these challenges, AMPs provide an alternative way to fight plant diseases and lessen dependency on traditional pesticides, changing the face of modern agriculture.

The future trend in antimicrobial peptide research lies in the synergistic integration of synthetic biology, nanotechnology, and precision agriculture to enhance the stability, specificity, delivery efficiency, and field applicability of antimicrobial peptides. Synthetic biology, utilising advanced genetic engineering tools such as CRISPR-Cas9, enables the large-scale production of optimised AMP variants with enhanced antimicrobial efficacy and proteolytic resistance. The nanotechnology-driven delivery platforms, such as liposomes, polymeric nanoparticles, micelles, hydrogels, and metallic nanostructures, provide protective, controlled-release, and targeted delivery mechanisms that enhance AMP bioavailability while minimising cytotoxicity. The incorporation of AMPs and nanoAMPs into precision agriculture and innovative farming systems allows environmentally sustainable PDM through transgenic expression and site-specific delivery, reducing chemical pesticide dependence and mitigating antimicrobial resistance. These interdisciplinary innovations are transforming AMPs from experimental molecules into effective, eco-compatible bioweapons for sustainable crop protection and global food security.

## 11 Conclusion

AMPs have emerged as an alternative to support the next-generation sustainable plant disease management. As endogenous members of innate immunity, AMPs show a safe, biodegradable, and pollution-free alternative to current synthetic pesticides as well as monitoring organic contamination and pathogen resistance problems. Broad-spectrum antimicrobial activities together with their multi-mechanism action contribute to effective control of hundreds of plant pathogens with reduced chances of resistance evolution. AMPs also promote plant growth, tolerance to abiotic stresses and crop productivity. Thus, they are not only important for plant-biotic interactions but are also vital for broad-spectrum agricultural sustainability. Novel peptide engineering, advances in synthetic biology and artificial intelligence-directed design, as well as genome editing technologies, including network biology, are further facilitating the rapid development of extremely stable, specific and field-usable AMP formulations. The advancements of these technologies, combined with industrial-scale production systems, delivery method optimisation, and corresponding regulations, support facilities towards real agricultural applications. Taken together, this evidence emphasises the developing potential for revolutionising strategies towards plant protection with AMP-based interventions. Thus, with the development and advancement of interdisciplinary research, AMPs are expected to ultimately play a role in the development of a sustainable crop protection system for resilient agriculture, reduced chemical reliance and sustainable global long-term food and environment security.
